# Theranostics Aspects of Various Nanoparticles in Veterinary Medicine

**DOI:** 10.3390/ijms19113299

**Published:** 2018-10-24

**Authors:** Ding-Ping Bai, Xin-Yu Lin, Yi-Fan Huang, Xi-Feng Zhang

**Affiliations:** 1 Fujian Key Laboratory of Traditional Chinese Veterinary Medicine and Animal Health, Fujian Agriculture and Forestry University, Fuzhou 350002, China; bdpnx@163.com (D.-P.B.); linazong1991@foxmail.com (X.-Y.L.); yfanhuang@163.com (Y.-F.H.); 2 College of Biological and Pharmaceutical Engineering, Wuhan Polytechnic University, Wuhan 430023, China

**Keywords:** nanoparticles, antimicrobial, diagnostic, animal production, livestock, drug delivery

## Abstract

Nanoscience and nanotechnology shows immense interest in various areas of research and applications, including biotechnology, biomedical sciences, nanomedicine, and veterinary medicine. Studies and application of nanotechnology was explored very extensively in the human medical field and also studies undertaken in rodents extensively, still either studies or applications in veterinary medicine is not up to the level when compared to applications to human beings. The application in veterinary medicine and animal production is still relatively innovative. Recently, in the era of health care technologies, Veterinary Medicine also entered into a new phase and incredible transformations. Nanotechnology has tremendous and potential influence not only the way we live, but also on the way that we practice veterinary medicine and increase the safety of domestic animals, production, and income to the farmers through use of nanomaterials. The current status and advancements of nanotechnology is being used to enhance the animal growth promotion, and production. To achieve these, nanoparticles are used as alternative antimicrobial agents to overcome the usage alarming rate of antibiotics, detection of pathogenic bacteria, and also nanoparticles being used as drug delivery agents as new drug and vaccine candidates with improved characteristics and performance, diagnostic, therapeutic, feed additive, nutrient delivery, biocidal agents, reproductive aids, and finally to increase the quality of food using various kinds of functionalized nanoparticles, such as liposomes, polymeric nanoparticles, dendrimers, micellar nanoparticles, and metal nanoparticles. It seems that nanotechnology is ideal for veterinary applications in terms of cost and the availability of resources. The main focus of this review is describes some of the important current and future principal aspects of involvement of nanotechnology in Veterinary Medicine. However, we are not intended to cover the entire scenario of Veterinary Medicine, despite this review is to provide a glimpse at potential important targets of nanotechnology in the field of Veterinary Medicine. Considering the strong potential of the interaction between the nanotechnology and Veterinary Medicine, the aim of this review is to provide a concise description of the advances of nanotechnology in Veterinary Medicine, in terms of their potential application of various kinds of nanoparticles, secondly we discussed role of nanomaterials in animal health and production, and finally we discussed conclusion and future perspectives of nanotechnology in veterinary medicine.

## 1. Introduction

In the present status of health care technologies, Veterinary Medicine will enter a phase of new and incredible transformations. Recently, nanotechnology offers great significant contributions for the development technology in human and Veterinary Medicine, particularly to create new knowledge and make it translation in to the field. Nanotechnology is one of the key technologies of the 21st century and it could offer numerous benefits to the human as well as animal for the developments various competitive devices in wide range of sectors, which would directly help human welfare. Nanotechnology is a multidisciplinary approach using principles of various subjects, including physics, chemistry, material science, biology, engineering, and medicine. Nanoparticle strictly refers to 1–100 nm in size [1]. Recently nanotechnology and nanomedicine offers significant contribution to clinical therapeutics using biocompatible nanoscale drug carriers, such as liposomes, micelle nanoparticles, dendrimer nanoparticles, polymeric nanoparticles, and metal nanoparticles for more efficient and safer delivery of various anticancer drugs. The nanoparticles mediated drug delivery provides longer circulation half-lives, improved pharmacokinetics, and reduced undesired side effects [2,3,4,5]. Nano-sized materials were employed to improve pharmacological therapies, novel modalities for the treatment and diagnosis. The new technology of nanomedicine increases the efficacy of delivery and potential in cancer treatment, particularly to decrease bio-distribution of a drug, thereby reducing off-target side effects, whilst increasing drug exposure to target cells only [6,7]. Nanoparticle based therapy could improve the balance between the efficacy and the toxicity of systemic therapeutic interventions [2]. Nanoparticles can carry a high dose of drug payloads; it would overcome the small molecules drugs, which are susceptible to transmembrane diffusion [8]. Nanomedicine offers several nanoparticulate platforms such as liposomes, liposome composites, lipid micelles, polymer micelles, polymer drug conjugates, dendrimers, protein carriers, biologically synthesized nanoparticles, and inorganic nanoparticles, as a drug delivery vehicle and some cases nanoparticles acting as cytotoxic and bio-imaging agents. Veterinary medicine is an important aspect taking care of health care in dairy industry to overcome the significant economic loss.

Domestic livestock are primary source for the living and getting revenue of more than 600 million farmers in developing countries and contribute to about 30–35 per cent of agricultural gross domestic product [9]. Devastating outbreaks of new diseases and re-emergence of old infections in animals lead to unbelievable loss of income for livestock keepers [10]. The delay of detection and controlling zoonoses is associated with spread of the vulnerable infection to entire herds and humans. Therefore, it is essential to explore the novel technology to prevent and treat the diseases that are caused by various microorganisms to the veterinary animals. The recent development in nanoscience and nanotechnology helps several areas of research and applications in Veterinary Medicine by developing new diagnostic tools and the development of new forms of treatments, which facilitate increasing the longevity and improve the quality of life of veterinary animals. Nanotechnology is specifically defined as the design, characterization, and application of structures, devices, and systems by controlling shape and size at the nanometer scale level (ranging from 1 to 100 nm) and nanomedicine considered to be offers a more strategic approach, these smaller size of particles can encapsulate and deliver drugs to dramatically enhance their effectiveness. Associating a therapeutic molecule with a nanoparticle can enhance its solubility by orders of magnitude, allowing for hydrophobic drugs to be carried more easily through the bloodstream. This could help to address a serious challenge in pharmacology, since an estimated 40 per cent of new drugs are poorly soluble in biological fluids [4]. In addition, nanoparticles can also enable the controlled release of the drug, or deliver two different drugs simultaneously to give a more powerful combination therapy. Most of the studies either beneficial or cytotoxic effects have been done on rodents as in vivo models due to the similarity in biochemical and physiological pathways with human metabolism, but limited studies are only available in veterinary animals [10]. However, the clinical studies were performed in both primary research focused on the treatment and diagnosis of veterinary diseases and translational research in which spontaneous diseases in animals can be used as models of human diseases. Based on the literature considering into account, this review focused on the basic principles behind the use of very commonly used nanoparticles for antimicrobial agents, drug delivery, diagnostics, vaccine formulation, feed additive, reproductive aides, animal growth, and animal production. Further, we discussed the clinical applications and limitations, providing the reader with a realistic synopsis of the practical applications of nanoparticles to veterinary medicine at present and in the near future.

## 2. Types of Various Nanomaterials Used in Veterinary Medicine

**Figure 1:**
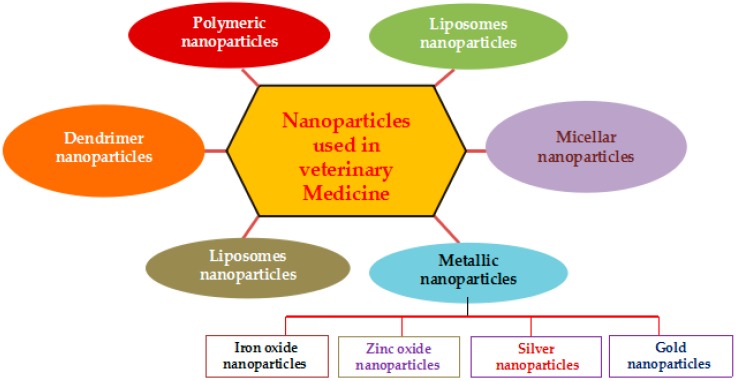
Type of nanoparticles used in application of veterinary medicine and animal production.

The variety of nanomaterials have been used for disease diagnosis, treatment, drug delivery, animal nutrition, animal breeding, reproduction, and value added products into animal products, including liposomes nanoparticles, micellar nanoparticles, polymeric nanoparticles, dendrimer nanoparticles, metallic nanoparticles, and carbon nanoparticles (Figure 1).

### 2.1. Liposomes Nanoparticles

Liposomes are small artificial closed spherical vesicles consisting of a lipid bilayer that encapsulates an aqueous phase in which drugs can be stored. The average size of liposome nanoparticle ranges from 100 to 2.5 mm. Liposomes are extensively used as carriers for numerous molecules in cosmetic and pharmaceutical industries due to its biocompatibility, biodegradability, low toxicity, and aptitude to trap both hydrophilic and lipophilic drugs, and it can deliver the drug to site specifically to tumor tissues [11,12]. Several practical applications of liposomes were developed by the pioneer work of Professor Greg Gregoriadis and collaborators [13,14], whereas Zolle et al. [15] developed albumin nanoparticles for numerous applications. Liposome mediated drug delivery offers improved pharmacokinetic properties, controlled and sustained release of drugs, less systemic toxicity, and precise drug delivery in cancer and which is best technology for multidrug resistant cancers [16]. The liposomes are composed of either synthetic or natural phospholipids. The physical and chemical properties of a liposome are controlled by the permeability, charge density, and steric hindrance of constituent phospholipids [17]. The special advantages of liposomes are that the physicochemical properties of liposomes can be precisely changed to control surface charge, functionality, and size by simply mixing commercially available lipid molecules. This offers a significant advantage over other carriers that require much more controlled synthesis steps and additional chemical modifications [18]. Although liposomes nanoparticles having variety of merits, still it has some challenges, such as instability in the bloodstream, poor solubility, burst release of drug, and severe side effects [19]. The efficacy of docetaxel is encapsulated into the liposome bi-layer was evaluated in human xenograft mice models for prostate, pancreatic, and non-small-cell lung cancer (NSCL cancer) showed partial tumor regression in 90% of the PC3 tumor xenograft model and improved efficacy in the pancreas model [20].

Recent advances in nanomedicine contributed a significant amount in the veterinary field and have found a wide variety of applications. Particularly, liposomes mediated drug delivery was much increased therapeutic substances in animals. Liposomes are nanosized phospholipid vesicles that can serve as delivery platforms for a wide range of substances [21]. Liposomes are easily formulated, highly modifiable, and easily administered delivery platforms. They are biodegradable and nontoxic and they have long in vivo circulation time [21,22]. Liposomes are divided into three types that are based on morphological characters, including multilamellar vesicles (500 to 5000 nm), large unilamellar vesicles (200 to 800 nm), and small unilamellar vesicles (100 nm). The liposomes mediated drug delivery was performed in dogs with canine splenic hemangiosarcoma (HSA) [21,22]. The results showed that liposome-encapsulated muramyl tripeptide enhanced antitumour potential and liposome-encapsulated muramyl tripeptide conjugated with phosphatidylethanolamine resulted in prolonged disease-free survival in the morbid canines [23,24]. Liposomes based vaccines were tested in various livestock, it exhibited strong immune responses against parasitic pathogens [25]. Liposomes have been used for targeted drug, imaging agent, vaccine, and gene delivery with promising results [25,26]. Nano-scale liposomes were formulated with different ratios of 1, 2-dimyristoyl-sn-glycero-3-phosphoethanolamine (DMPE), dihexadecyl phosphate (DCP), and cholesterol (Chol): DMPE, DMPE/DCP, DMPE/Chol, and DMPE/DCP/Chol were produced. The immunological responses were examined by using the newly formed nano-liposomes via porcine interferon gamma (IFN-γ) enzyme-linked immunospot (ELISPOT) assay in peripheral blood mononuclear cells (PBMC) of immunocompetent pigs. The porcine IFN-γ ELISPOT assay results revealed that DMPE/DCP most potently induced the IFN-γ secretion by PBMC, followed by DMPE/DCP/Chol and DMPE [27]. Liposomes based on phospholipids used as adjuvant and adjuvant carrier in various microbial diseases caused by different type of microorganisms, including *P. malarie* [28], Influenza Virus (INFLEXAL^®^ V) [29], HAV (Epaxal^®^) HIV [30], and ICMV P. vivax [28]. Intraocular immunization with liposome-associated fimbrial antigens resulted in significant increases in IgA and IgG profiles, along with counts of antibody-producing lymphocytes in various livestock [31]. Malam et al. demonstrated detailed information about liposomes and nanoparticles as drug delivery agents [16].

### 2.2. Therapeutic Applications of Liposomes to Control Microbial Diseases in Veterinary Medicine

The target of human and veterinary medicine is first the treatment and prevention of diseases. In both cases, liposomes contributed major part in controlled release and targeted to specific sites without any side effects using liposomal formulations. Sallovitz et al. [32] described very extensively about bacterial diseases in human and veterinary medicine. Liposomal formulations have been addressed in several bacteria including *Staphylococcus aureus*, *Salmonella* species, *Brucella* species, and *Mycobacterium* species [33]. MacLeod and Prescott demonstrated that liposomally entrapped gentamycin was used to kill *Staphylococcus aureus* mastitis in bovine [34]. Liposomes containing dipalimitoyl phosphatadylcholine, and dimyristodylphosphatadyl glycerol encapsulating tobramycin showed considerable antibacterial effects against Gram positive and negative bacteria [35,36]. The nisin-based formulation showed better efficacy in the treatment of clinical mastitis in lactating dairy cows caused by several different mastitis pathogens on a commercial dairy farm [37]. Liposomes entrapped phage shows effective targeting against multidrug resistant bacterial infections [38]. The causative agent of chronic pulmonary bacterium called *Mycobacterium avium* intrinsic resistance conventional chemotherapeutic agents, however when treated with aminoglycosides shows more effective [39]. 

Fungi are known to cause diseases, including candidiasis, cryptococcosis, and histoplasmosis. Liposomal AmB (LAmB) is known to be effective and less toxic than conventional antimicrobial agents, which was demonstrated in several animal models [40,41]. Krawiec et al. demonstrated the high efficacy of LAmB in dogs infected with various degree of severity of blastomycosis [42]. The treatment of fungal pneumonia is tedious process and it is very difficult to treat animals. However, when the animals administered to LAmB showed more efficient and reduced level of disseminating [40]. The incorporation of photosensitizers (PSs) into liposomes, micelles, or nanoparticles seems to alternative and it is a promising approach to reduce the PS self-aggregation and to enhance the targeted delivery of the PS in various microbial diseases [43]. Khan et al. formulated liposomes containing amphotericin B in tuftsin showed enhanced efficacy against systemic cryptococcosis in leucopenic mice [44].

Viral diseases are the major source of morbidity, mortality, and economic loss in human and animals. Liposomes are reasonably increasing the drug efficacy without inducing toxicity to the host cells; it could target only replication of virus by delivering the drugs at specific sites. Liposomal entrapped ribavarin and 2’3’-dideoxycitidine have shown promising formulation for viral diseases [45,46]. Rhee et al. formulated hemagglutinin-derived synthetic peptide with a CpG-DNA-liposome complex, which induce the protection against lethal influenza virus [47]. The toxicity of a novel liposome formulated of meglumine antimoniate in dogs with visceral leishmaniasis, the results indicated that the newly formulated compounds exhibited significant cytotoxicity [48]. Sadozai and Saeidi dedicated exclusive review on recent developments in Liposome-based veterinary therapeutics [21]. 

### 2.3. Micellar Nanoparticles

Micellar nanoparticle was initially developed in the mid-1990s [49,50,51]. Micellar nanoparticles are frequently used in in transdermal therapeutics [52]. Micellar nanoparticles are a vigorous and versatile delivery system that can accommodate a range of therapeutic compounds having varying physicochemical properties [52]. Micellar nanoparticle mediated drug delivery offers a potentially fast, cost effective and higher loading capacity for using drugs. Micellar nanoparticle having with an average size between 8 and 125 nm and it can accommodate both water-soluble and poorly water-soluble active pharmaceutical ingredient (APIs). MNPs (Magnetic Nanoparticles) are composed of APIs; solvent; stabilizer; oil; and aqueous medium. Particularly the studies related to veterinary animals are very countable. For instance, Scott-Moncrieff et al. (1994) conducted to examine insulin uptake by micelles with bile-salty fatty acids in dogs [53]. Insulin combined with mixed micelles is entirely absorbed in dogs; however, the bioavailability is much lower than that observed in similar studies with rats. The comparative study was performed to determine biodynamic parameters of aqueous and water-disperse diminazene micellar in sheep erythrocytes and plasma in sheep. The study found that the surface-active substances improved intracellular penetration of the active substance through interaction with the cell membrane. In sheep blood erythrocytes, micellar diminazene accumulated more than its aqueous analog. This form was also more effective therapeutically than the aqueous analog. The study concluded that the use of micellar diminazene allows for the injection dose better in aqueous than water [54]. A randomized trial was conducted to determine the efficacy and safety of water soluble micellar paclitaxel (Paccal Vet) compared with free lomustine for treatment of nonresectable grade 2 or 3 mast cell tumors in dogs. The results exhibited that paclitaxel (micellar)’s activity and safety profile are superior to lomustine [55]. The oral supplementation of micellized natural vitamin E in race horses efficiently increased plasma α-tocopherol concentration than control, which suggests that the supplementation of micellized natural vitamin E maintained the general oxidative status of race horses under intense trainin [56]. Next study, the same authors conducted in adult and weaning piglets to determine the oxidative status by using lower oral doses of micellized α-tocopherol compared to α-tocopheryl acetate in feed modify fatty acid profiles. The study concluded that oral supplementation of sows (75 mg/day) and piglets (1.7 mg/day) with micellized natural vitamin E modified the fatty acid profile of piglet tissues and improved their oxidative status [57]. The bioavailability and pharmacokinetic parameters of tilmicosin, which is semisynthetic antimicrobial agent was supplemented to broiler chickens via oral administration using three different micellar nanoparticles, including solid lipid nanoparticles, nanostructured lipid carriers, and lipid-core nanocapsules. Among three different nanoparticles, tilmicosin that was prepared with lipid nanoparticles improved drug bioavailability and pharmacokinetic parameters in broiler chickens [58]. Troncarelli et al. described the importance of various nanoparticles as anti-microbial agent in veterinary medicine [59].

### 2.4. Polymeric Nanoparticles

Polymeric nanoparticles are derived from polymers, which are macromolecules that are composed of a large number of repeating units organized in a chain-like molecular architecture exhibiting a multiplicity of compositions, structures, and properties [60]. Due its compositions, structures, and properties that polymers are being used in nanoparticle systems to generate nanoparticles that are suited for each specific biomedical application. Polymeric nanoparticles are mainly used in drug delivery [60]. The following features are the main reason for the selection of polymer–drug conjugates as compared to the parent free drug, which has the ability for passive tumor targeting by the enhanced permeability and retention effect, decreased toxicity, increased solubility in biological fluids, ability to overpass some mechanisms of drug resistance, and finally ability to elicit immuno-stimulatory effects [3,61,62]. Both natural and synthetic polymeric nanoparticles have been used for the delivery of oligonucleotides, DNA, and protein and drugs. For instance, natural polymer, such as an albumin-paclitaxel nanoconjugate, was used for the treatment of metastatic breast cancer during phase III clinical trials [63]. Synthetic polymers for nanoparticles, such as *N*-(2-hydroxypropyl)-methacrylamide, copolymer (HPMA), poly(ethylene glycol) (PEG), poly (lactic acidglycolic acid) (PLGA), and poly(lactic acid) PLA were used as drug delivery agents both in vitro and in vivo studies [64]. Polymer-based nanoparticles being used in skin administration because of its controlled release. For example, Hydrogel (Carbopol^®^ Ultrez 10 National Formulatory) containing dexamethasone as the active ingredient has shown potential use in controlled drug delivery for the treatment of psoriasis [65]. Nanoencapsulated siRNAs have been used for the management of pachyonychia congenital and for successful targeted delivery and inhibition of a test gene expressed in melanoma in human trials [66]. Synthetic polymeric nanoparticles, such as PLG, PLGA, PGE, Polystyrene, and Polyester Bio-Beds nanoparticles were used to target Toxoplasmosis and HIV, *S. aureus*, TB, *Brucella abortus*, antrhrax, *Plasmodium vivax*, HBV, Influenza Virus, HIV, *P. malariae*, TB, respectively [28,67,68,69,70,71,72,73,74,75]. Similarly natural polymeric nanoparticles were used in different diseases, including Inulin: ADVAX™ in Anthrax [76]; Listeria monocytogenes [77], Influenza virus [78], SARS-CoV [79], HBV [80], HIV, and JVE-WNV [81]. Alginate and pullulan composed nanoparticles used in various microbial diseases that are caused by *Klebsiella pneumoniae* [82], *Pseudomonas aeruginosa* [83], and Influenza virus [84], Difteria [85]. Similarly polymeric nanoparticles composed of chitosan were used in various microbiological diseases caused by *E. coli* O157:H7 [86], *P. aeruginosa* [87], Influenza virus [74], HBV [88], Filariasis [89], and Dengue [90]. Recently, Hill and Li demonstrated that the application of various nanoparticles in animal production [91]. Polymeric nanoparticles are being utilized for drug loading depends on characteristic features of polymeric nanoparticles, such as biocompatibility, targeting, degradation, and controlled release kinetics [92].

### 2.5. Dendrimer Nanoparticles

Dendrimers are highly branched, well defined, organic compounds with well-defined, regularly hyper branched and three-dimensional architecture and symmetrical structure. In addition, dendrimers are relatively low polydispersity, high and tunable functionality and molecule properties are significantly different than the linear counterparts [93]. The first family of dendrimers was the poly (amidoamine)s (PAMAM) [94]. The physical and chemical properties of dendrimers are mainly influenced by the structure. Dendrimers have been used extensively in biomedical applications and veterinary medicines due to cost effective, stability, monodisperse and controllable size, modifiable surface, functionality, multivalency, water solubility, high transfection efficiency, and the ability of dendrimers to penetrate the cell membrane [95,96]. Furthermore, the availability of numerous functional groups on the surface of particles enables the attachment of multiple copies of both the identical and the different antigens, and therefore enhances the immunogenicity of the vaccines [97]. Dendrimer based nanocomposites showed efficient antibacterial activity against various Gram positive and Gram negative bacteria. Synthetic oligonulceotides and antigens in biodegradable nanospheres were used for immunization. Dendrimers were demonstrated as promising tools for MRI imaging, gene transfer, and as antimicrobial agents [98,99]. Dendrimers offers various advantages in veterinary medicine for the transport of drugs as vehicle for various agents (e.g., genes and anticancer drugs) by complexation or encapsulation [100], to improve the pharmacokinetic behavior of currently available small-sized compounds from a broad extracellular to an intravascular distribution [101]. Further, dendrimers are frequently used in angiography, tissue perfusion determination, tumor detection, and differentiation [102]. Xiao et al. developed bioactive cell-like hybrids by the assembly of (glyco) dendrimersomes with bacterial membranes [103]. These hybrid vesicles contain transmembrane proteins, including a small membrane protein, MgrB, tagged with a red fluorescent protein, lipopolysaccharides, and glycoproteins from the bacterium Escherichia coli. These hybrid vesicles will be used for practical applications in human and veterinary animals. Samad et al. dedicated exclusive review on synthesis, characterization, and recent applications of dendrimers, particularly the clinical usage of dendrimers [104].

### 2.6. Metallic Nanoparticles

Metallic nanoparticles are nanosized metals with an average size between 1 and 100 nm. Under this definition, one can classify metallic nanomaterials in to four main categories. They are classified into four types, including metallic nanoparticles (0D), metallic nanowires and rods (1D), metallic sheets and platelets (2D), and metallic nanostructures (3D). Metallic nanoparticles show much interest in industry and academic due its physical and chemical properties; currently it has several applications in biotechnology, biomedical science, engineering, and nanomedicine [105]. The metallic nanoparticles can be synthesized and modified with appropriate functional groups that would allow them to bind with drugs, antibodies, ligands: substances of high interest in biomedical field [106]. Presently, antibiotic resistance in bacteria reached to been reaching a life-threatening level. At this juncture, exploring various options to address this problem, inorganic nanomaterials, like metal oxide nanoparticles, have emerged as promising candidates, since they possess greater durability, lower toxicity, and higher stability and selectivity when compared to organic ones [107]. Metal nanoparticles are mostly used in bio-sensing, bio-imaging and cancer thermotherapy by conjugating with various functional groups and yielding stable nanoparticles within the range beween 1 and 100 nm [108]. The use of selective nanoparticles in veterinary Medicine is discussed, as follows.

### 2.7. Silver Nanoparticles

Recently, silver nanoparticles (AgNPs) shows much interest in biomedical applications including diagnosis, treatment, drug delivery, medical device coating, wound dressings, textiles, and contraceptive devices as an antibacterial, antiviral, antifungal, anti-inflammatory, anti-cancer, and anti-angiogenesis [105,109]. The application of nanoparticles in veterinary medicine and animal production have been used as sensors, imaging, drug delivery, and tissue engineering, however the application of AgNPs in veterinary medicine is limited [110]. Biologically synthesized AgNPs with an average size of 60 nm with concentrations of 15 and 20 mg/kg of AgNPs-loaded vaccine injected into the mice and dogs. The in vivo toxicity also elucidated the safety of AgNPs and AgNPs-loaded vaccine in mice and dogs, respectively. Safety test for veterinary use was carried out on two healthy female dogs. After administration of AgNPs-loaded rabies vaccines, the animals were daily monitored for 14 days. The animals did not show signs of either disease or local and systemic reactions and no fever or death was observed due to the vaccine during monitoring. The study shows that AgNPs can be used as adjuvant in rabies veterinary vaccine. AgNPs interact with the virion surface as well with the virion core Peste des petits ruminants virus and it shows that there is no significant effect on the virucidal effect, instead it exerts a blocking effect on viral entry into the target cells [111]. The antiviral activity of tannic acid modified various sizes of AgNPs was studied. The study concluded that it required direct interaction and blocked virus attachment, penetration, and further spread [112].

**Figure 2:**
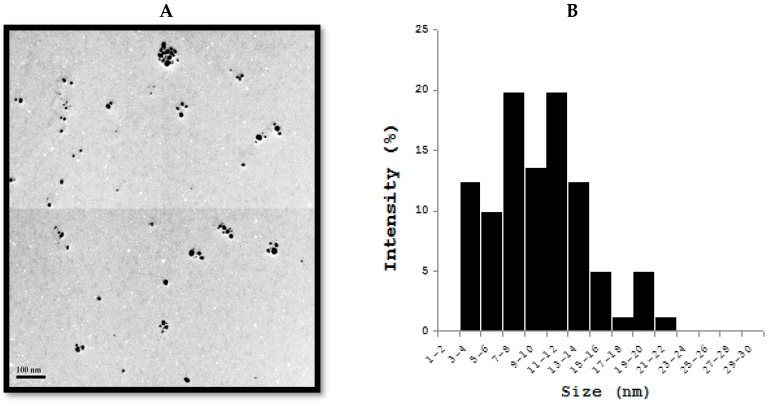
Transmission electron microscopy (TEM) of silver nanoparticles (AgNPs) (**A**) TEM images of AgNPs synthesized by culture supernatant of *Bacillus marisflavi* (**B**). Size distribution of AgNPs from TEM images.

**Figure 3:**
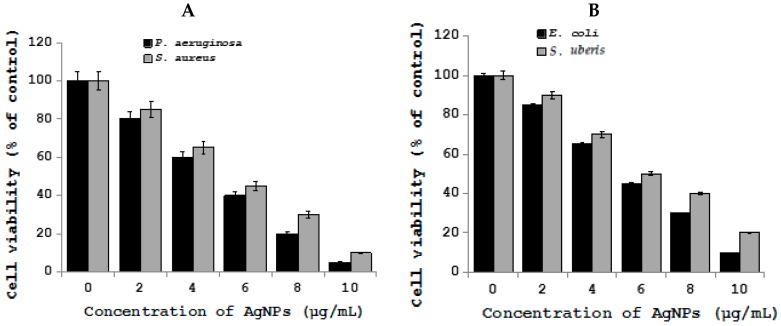
Effect of silver nanoparticles on *S. aureus* and *P. aeruginosa*, *E. coli,* and *S. uberis* from mastitis-infected goats. (**A**) Cell viability of *S. aureus* and *P. aeruginosa* treated with AgNPs. (**B**) Cell viability of *E. coli,* and *S. uberis* treated with AgNPs. Bacterials cells were incubated with various concentrations of AgNPs. Bacterial survival was determined at 24 h by a CFU (colony forming unit) count assay. The experiment was performed with various controls, including a positive control (AgNPs and NB, without inoculum) and a negative control (NB and inoculum, without AgNPs). The results are expressed as the means ± SD of three separate experiments, each of which contained three replicates. Treated groups showed statistically significant differences from the control group by Student’s *t* test (*p* < 0.05).

Antibiotic resistant bacteria are a serious health risk in both human and veterinary medicine. Therefore, developing combined therapy contains low levels of antibiotics and AgNPs to enhance the antibacterial effect is a novel approach [113,114]. Several studies have shown that AgNPs exert a high level of antibacterial activity against antibiotic resistant strains in humans. The evaluation of antibacterial effects of a combined therapy of AgNPs and antibiotics against veterinary bacteria is limited. The majority of synergistic effects were observed for combinations of AgNPs given together with gentamicin, but the highest enhancement of antibacterial activity was found with combined therapy together with penicillin G against *Actinobacillus pleuropneumoniae* [115]. Recently, Yuan et al. demonstrated the effects of AgNPs on multiple drug-resistant strains of *S. aureus* and *P. aeruginosa* from mastitis-Infected goats [116]. Metallic AgNPs are able to reduce the viability of potential harmful coliform bacteria, whereas there is no harmful effect in Lactobacilli in ileal contents [117]. Fondevila et al. studied the ileal contents of weaned piglets given AgNPs (20 and 40 ppm), the results clearly indicated that the amount of coliform bacteria was significantly reduced [118]. When piglets given 25 ppm of AgNPs did not observe any significant reduction of coliform bacteria but it increases the amount of lactic acid bacteria [119]. One-year-old female intact American Staffordshire Terrier with 50% total body surface area burned was successfully treated by nanocrystalline silver dressing and vacuum-assisted closure [120]. To investigate the effect of AgNPs on bacteria causing mastitis in goats, we synthesized AgNPs using *Bacilllus marisflavi* cellular extract, the prepared AgNPs shows significantly spherical in shape with an average size of 20 nm (Figure 2) [121]. Bacteria called *S. aureus* and *P. aeruginosa* treated with AgNPs exhibited significant antibacterial activity (Figure 3) [122]. Nanosilver effect was evaluated in the reduction of aflatoxin on the growth and performance indices in broiler chickens suffering from experimental aflatoxicosis. When the supplementation of silver with a conventional diet had no effect on performance, but the addition of nanosilver to diet containing 3 ppm aflatoxin increased significantly the cumulative BWG, cumulative feed consumption, and decreased FCR in the last two weeks of the experimental period. The study concluded that supplement of nanosilver to diet containing aflatoxin could diminish the inhibitory effects of aflatoxin [123]. Similarly, the same authors further demonstrated that nanosilver, as mycoad, can be useful in reducing the adverse effects of aflatoxin on blood parameters in chickens that are affected with aflatoxicosis [124]. The combination of nanocrystalline silver and subatmospheric pressure therapy shows significant effect in cat after following tumour removal and radiation therapy [125].

The study was performed to determine the effect of AgNano as an antimicrobial growth-promoting supplement for broiler chickens via drinking water. The water solutions containing different concentrations of AgNano had no effect on postnatal growth performance and the energy metabolism of broiler chickens. Interestingly, the concentration of immunoglobulin (IgG) in the blood plasma of broilers supplemented with AgNano decreased at day 36. However, it does not influence the microbial populations in the digestive tract, the energy metabolism, and growth performance of chickens [126]. In Ovo administration of AgNPs and/or amino acids was performed to determine the effect on metabolism and immune responses in chicken embryos. The results concluded that NanoAg either alone or in combination with amino acids did not affect embryonic growth but improved immuno-competence, indicating that NanoAg and amino acid complexes can act as potential agents for the enhancement of innate and adaptive immunity in chicken [127]. The nano-functionalized antimicrobial oils were used in the formulation of shampoo, soap, and ointment for veterinary dermatology. The nano-functionalized oil showed remarkable antimicrobial activity against pathogens that are present on the skin of animals; therefore, it is valuable, safe, and has a specific role in controlling diseases [128]. Biologically synthesized AgNPs were used as adjuvants in rabies veterinary vaccine with the existing commercially available alum adjuvant and the toxicity was evaluated in mice and dogs. The results showed that the adjuvanticity effect of green synthesized AgNPs on veterinary rabies vaccine potency found to be no toxicity [129]. The effect of AgNPs pleural effusions in exposed factory workers while in experimental animal studies possible to induce inflammation, fibrosis, and carcinogenesis in the pleura. Therefore, the membrane permeability of sheep parietal pleura, of primary sheep pleural cell monolayers, and on a human mesothelial cell line was undertaken. The results from this study concluded that acute (30 min) exposure increases the pleural permeability ex vivo, while longer (24 h) in vivo exposure leads to a late decrease of the pleural cell monolayers permeability [130]. Recently, Gurunathan et al. demonstrated that antibacterial potency of AgNPs on endometritis causing *Prevotella melaninogenica* and *Arcanobacterum pyogenes* in Dairy Cattle by inhibition of cell viability and biofilm formation in a dose- and time-dependent manner [131]. Moreover, the metabolic activity was blocked by AgNPs by the increased generation of reactive oxygen species (ROS), malondialdehyde, protein carbonyl content, and nitric oxide.

### 2.8. Gold Nanoparticles

**Figure 4:**
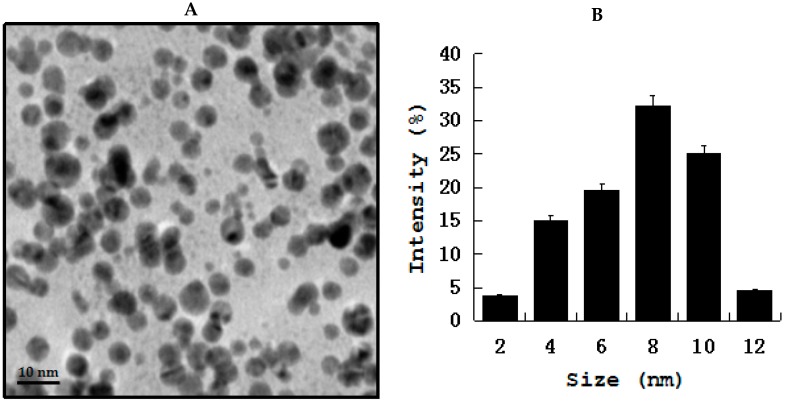
(**A**) TEM images of AuNPs (**B**). Size distribution of AuNPs from TEM images. TEM images of several fields were used to measure AuNPs particle size; micrographs (left panels); and, size distributions based on TEM images (right panels) of AuNPs ranging from 2 nm to 12 nm.

**Figure 5:**
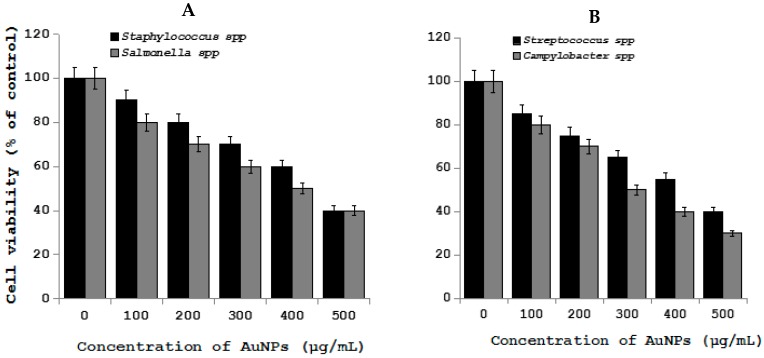
Effect of AuNPs on *Staphylococcus* spp., *Salmonella* spp., *Streptococcus* spp., and *Campylobacter* spp. in chicken. *Staphylococcus* spp., *Salmonella* spp., *Streptococcus* spp., and *Campylobacter* spp. cells were incubated with various concentrations of AuNPs. (**A**) Cell viability of *Staphylococcus* spp. and *Salmonella* spp. treated with AuNPs. (**B**) Cell viability of *Streptococcus* spp. and *Campylobacter* spp. treated with AuNPs. Bacterial survival was determined at 24 h by a CFU count assay. The experiment was performed with various controls, including a positive control (AuNPs and NB, without inoculum) and a negative control (NB and inoculum, without AuNPs). The results are expressed as the means ± SD of three separate experiments, each of which contained three replicates. Treated groups showed statistically significant differences from the control group by Student’s *t* test (*p* < 0.05).

Gold nanoparticles (AuNPs) show immense interest in biomedical applications due its several advantageous properties, including a non-toxic and biocompatible metal [132]. Furthermore, AuNPs have the ability to undergo multiple surface functionalization combining different moieties, such as drugs and targeting agents, which renders them a highly versatile tool for targeting [133]. Interesting feature of AuNPs are imaging modalities for in vitro and in vivo cell tracking, which is valuable imaging agents for diagnoses and therapeutics. Recently, AuNPs have been used extensively in clinical applications, including biological sensing, biomedical imaging, drug delivery, and photo-thermal therapy due its unique physical, chemical, biocompatibility, and biological properties. Gold nanoparticles and gold-based test strip have been used for the rapid detection of infectious bursal disease virus antibodies in chickens [134], foot-and-mouth disease virus [135], pathogenic bacteria [136], bluetongue virus [137], specific bacterial contaminants in chicken, such as *S. typhimurium* and *S. enteritidis* [138], antibacterial effect against Bacillus Calmette-Guérin (BCG) and *Escherichia coli* [139], haptoglobin in mastitic milk of bovine [140], detection of *Melissococcus plutonius*, the causative agent of European foulbrood [141], determination of clenbuterol in bovine hair samples [142], diagnosis of viral infections in pigs [143], rapid detection of Mycoplasma suis in porcine plasma [144], bacterial toxins [145], as diagnostic tool for the serodiagnosis of cystic echinococcosis [146], Porcine circovirus type 2 [147], rapid determination of *Escherichia coli* O157:H7 in minced beef and water [148], serological detection of *Toxoplasma gondii* infection in dogs and cats [149], and the determination of five quinoxaline-1,4-dioxides in animal feeds [150]. To determine the cell viability effect of gold nanoparticles on pathogenic bacteria in chicken, such as *Staphylococcus*, *Salmonella* spp., *Streptococcus*, *Campylobacter* spp. AuNPs was prepared using biological template and then prepared AuNPs found to be 20 nm (Figure 4) The effect of AuNPs was moderate when compared to silver and Zinc oxide nanoparticles (Figure 5). 

Gum arabic- (GA) and maltose- (MALT) stabilized AuNPs were administered intravenously to juvenile swine, and blood, tissue, and urine samples were collected and then analyzed for gold analysis. From this, the study concluded that AuNPs have an important role in determining the tissue distribution profile for individual AuNP constructs [151]. AuNPs used as X-ray contrast agent for tumor imaging in mice and dog [152]. Gum arabic-coated radioactive gold nanoparticles (GA-(198) AuNPs) that were used for the treatment of prostate cancer in canine. GA-(198) AuNPs injected intralesionally and then evaluated for toxicity and effectiveness. The results showed that GA-(198) AuNPs immediately enter into the region of prostate and hematologic or biochemical parameter shows there is no significant difference between treated and untreated. This study concluded that AuNPs could be used as therapeutic agent for veterinary animals [153]. According to several studies in human cell lines AuNPs seems to be non-toxic and use noble metal nanocolloids as alternative medicine, however it depends on concentration used for test. Recent study from porcine T lymphocytes and on IL-2 and IL-10 synthesis in porcine peripheral blood mononuclear cells, the nanocolloid was not cytotoxic to porcine leukocytes, nor did it affect resting T cells. However, high nanogold concentrations inhibited proliferation of mitogen-stimulated CD4+, CD4+CD8α+, and CD4-CD8α- T cells by down-regulation of the IL-2 synthesis and increased the percentage of CD4-CD8α- double negative T cells, probably by depressing their ability to express a CD8α marker after activation. It suggests that AuNPs have potential immunosuppressive activity, which can strongly influence CD4-CD8α- T cells, which is the most abundant subpopulation in young animals. Recent studies from Mohamed et al. showed that antibacterial activity of AuNPs against *Corynebacterium pseudotuberculosis*, which is the etiological agent of chronic caseous lymphadenitis and it causes major economic losses due to major infection in goats and sheep [154]. 

### 2.9. Iron Oxide Nanoparticles

Murine model that was exposed to iron oxide nanoparticles exhibited significant attenuated inflammatory reactions that are associated with DTH, including the footpad swelling, the infiltration of T cells and macrophages, and the expression of interferon-γ, interleukin-6, and tumor necrosis factor-α in the inflammatory site in a dose-dependent fashion, significantly, and also iron oxide nanoparticles also demonstrated a suppressive effect on ovalbumin-stimulated production of interferon-γ by splenocytes and the phagocytic activity of splenic CD11b+ cells [155]. Canine adipose-derived stem cells were incubated with superparamagnetic iron oxide nanoparticles (SPIO: 319.2 μg/mL Fe) for 24 h. The iron oxide nanoparticles enter into the cells via endocytosis. Further the results showed there is no significant harmful effect on the viability of adipose-derived canine mesenchymal stem cells; therefore, it can be used for as contrast agent [156]. Long et al. reported that ultra-small superparamagnetic iron oxide nanoparticles used as MRI tracking agent to measure the seizure disorders in a rat model of temporal lobe epilepsy [157]. Recently, superparamagnetic iron oxide nanoparticles were used for cell-tracking method in an ovine model of tendonitis for the presence and distribution of intralesional cells in sheep. Histological studies revealed that after 14 days treatment SPIO particles remained embedded in tissue, providing an MRI signal and it seems to be SPIO labeling was an effective method for cell tracking for a large animal model of tendon injury for up to seven days post-injection [158]. Edge et al. studied dimercaptosuccinic acid (DMSA) coated superparamagnetic iron oxide nanoparticles for the medical applications, including diagnosis and targeted treatment of cancer using large animal, such as pigs as a model [159]. They analyse the pharmacokinetic, bio-distribution, and biocompatibility results revealed that magnetic nanoparticles (MNP) accumulation was observed primarily in the liver and spleen using MRI scans. Therefore, MNP can be used as diagnostic and therapeutic agents.

### 2.10. Zinc Oxide Nanoparticles

**Figure 6:**
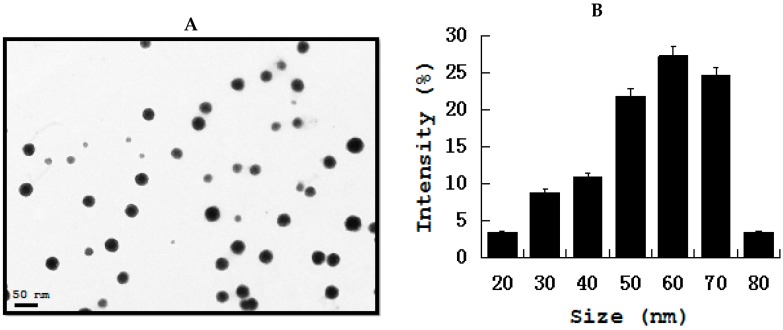
TEM of ZnO-NPs (**A**) TEM images of ZnO-NPs (**B**). Size distribution of ZnO-NPs from TEM images.

**Figure 7:**
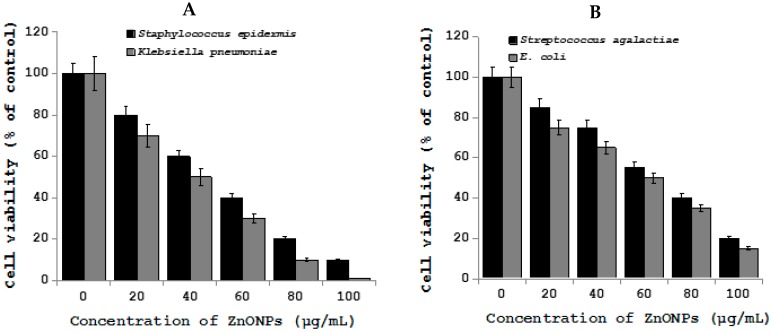
Antibacterial activity of ZnO-NPs on *Staphylococcus epidermis*, *Klebsiella pneumoniae*, *Streptococcus agalactiae* and *E. coli*. *Staphylococcus epidermis*, *Streptococcus agalactiae*, *Klebsiella pneumoniae*, and *E. coli* cells were incubated with various concentrations of ZnO-NPs. (**A**) Cell viability of *Staphylococcus epidermis* and *Klebsiella pneumoniae* treated with ZnO-NPs. (**B**) Cell viability of *Streptococcus agalactiae* and *E. coli* treated with ZnO-NPs. Bacterial survival was determined at 24 h by a CFU count assay. The experiment was performed with various controls including a positive control (ZnO-NPs and NB, without inoculum) and a negative control (NB and inoculum, without ZnO-NPs). The results are expressed as the means ± SD of three separate experiments, each of which contained three replicates. Treated groups showed statistically significant differences from the control group by Student’s *t* test (*p* < 0.05).

The metal oxide nanoparticles, particularly Zinc oxide nanoparticles are most commonly used in catalysis, sensors, and environmental remediation and personal care products [160]. Recently, ZnO-NPs that were used in various applications of veterinary sciences due to their antibacterial, antineoplastic, wound healing, and angiogenic properties, and further ZnO-NPs have been used in tissue repair, as food preservative and as feed additive. ZnO-NPs found to inhibits the viability wide range of bacteria by strong interaction with bacterial cells and it could induce microbial cell injury by the generation of hydrogen peroxide from the surface of ZnO and finally it can enter into the bacteria by interacting with phosphorus and sulphur containing compounds, like DNA of bacteria [161,162,163,164]. Mastitis is a disease of high yielding animals that is commonly caused by *Staphylococcus*, *Streptococcus,* and *E. coli*, which leads to economic consequences by reducing the yield of milk. To treat various causative agents of mastitis the usage of antibiotics has been increased and eventually leads to antibiotic resistance [165,166]. ZnO-NPs have been found to be effective against biofilm inside the udder tissue causative agents, such as *S. aureus* and *E. coli* [167,168]. Arabi et al. found that the bactericidal effects on both Gram-positive and Gram-negative bacteria and also effective against spores that are resistant to high temperature and high pressure by the mechanism of increase the permeability and inhibition of membrane transport and eventually leads to cell death [169,170,171]. To determine the effect of Zinc oxide nanoparticles on various mastitis causing bacteria, including *Staphylococcus epidermis*, *Streptococcus agalactiae*, *Klebsiella pneumoniae*, and *E. coli*, the prepared ZnO-NPs with an average size of 20 nm (Figure 6). While the pathogenic bacteria treated with ZnO-NPs shows that pronounced decreased level of cell viability in all the tested bacterial strains (Figure 7).

The peculiar properties of ZnO NPs such as selective toxicity of preferential killing of cancer cells with minimal toxicity to normal primary immune cells and cancer cells, it can be used anti-cancer agent in both humans and veterinary animals [172], and also it is more sensitive to detect cancer biomarkers, therefore it can be easily used in ZnO-NPs based diagnostic devices. Due to all these, salient features of ZnO-NPs leads to significant usage in diagnostic and therapeutic purposes in common neoplastic conditions of animals, like lymphoma, cutaneous cancer, transmissible veneral tumor, and equine sarcoids [160]. Recently, neoplastic disorder was most frequently found in in domestic animals, for example, haematopoietic tumours, canine transmissible veneral tumor in canines, and equine sarcoid is the most common fibroblastic skin tumor affecting horses, mules, and donkeys. These neoplastic conditions could be treated by ZnO NPs [173,174,175,176,177,178,179]. Zinc oxide was used as feed additive in weaner piglets to overcome the effect of post-weaning diarrhoea (PWD) caused by enterotoxigenic *E. coli*, which causes an increase in morbidity and mortality and decrease growth rate during the weaning period in piglets [180]. Similarly, the addition of zinc (zinc oxide) at the concentration of 2500 to 3500 ppm in feed modulated the microbial status of the digestive tract and reduced the incidence of post-weaning diarrhoea in piglets and increased productive performances [181,182,183,184]. Zn is mainly used in human and livestock foods and feeds for normal physiological functions, as well as to meet the daily requirement [185]. Salama et al. studied the effects of dietary supplements of zinc-methionine on milk production, udder health and zinc metabolism in dairy goat, the results showed that addition of Zn enhanced resistance to udder stress in dairy goats to Zn supplementation [186]. When supplemented to poultry leads to increase the level of ADFI, ADG, DM, and intramuscular fat contents of the breast muscle, percentage of eviscerated yield, redness value in breast muscle, and pH values in thigh muscle and decreased shear force in thigh muscle, drip loss in breast and thigh muscle [187]. ZnO-NPs enhanced growth performance, improve feed utility and provide economic benefits in weaning piglets and growth, production, and dress performance in poultry [188,189,190]. The supplementation of increased doses of Zn up to 3000 mg/kg increases the growth and reduces diarrhoea in pigs [191]. Rajendran et al. found that the application of Nano Zn reduced the level of the somatic cell counts in cows with subclinical mastitis and improve milk production when compared with other conventional ZnO sources [192]. Zn deficient diets are a cause of high incidence of abortions and stillbirths and supplementation in the form of Zn nanoparticle to animals increase the reproduction [193,194].

The beneficial effects and toxic effects of any nanomaterials depend on size, shape, surface charge, dose response, aggregation, type of solvent, and type of host cells. ZnO-NPs not only causes beneficial effects but also causes the toxicity effect in several veterinary animals. ZnO-NPs causes severe toxicity in the pancreas; kidney, liver, rumen, abomasum, small intestine, and adrenal gland were observed in sheep [195]. Liver, spleen, heart, pancreas, and bone are the target organs of ZnO-NPs on oral exposure [196]. Najafzadeh et al. observed the mild liver toxicity and severe renal damage in lambs [194].

### 2.11. Carbon Nanoparticles (CNPs)

Carbon nanoparticles, such as nanodiamond, graphite, graphene oxide, fullerenes, carbon nanotubes and carbon nanohorns, and so on have been widely studied and utilized for biomedical applications, such as diagnostic and therapeutic, due its biocompatibility and low toxicity. However, their impact on an organism with prolonged exposure with higher concentration is not known. CNPs are extensively used as drug carriers [197]. However, single walled carbon nanotubes trigger oxidative stress and are cytotoxic in cultured cell lines [198] and toxicity and biodegradability are a significant concern in clinical application [199,200].

Nanodiamonds are an allotropic form of carbon whose core consists of the sp^3^ orbital crystal structure and the sp^2^ phase forms most of their surface. Interestingly, it has unique characteristics, such as hardness, fine-tuning of their size distributions, emission of strong fluorescence, color centers, such as nitrogen vacancy (NV)-centers, photostability, chemical stability, small size, large surface area, high adsorption capacity, and good biocompatibility [201,202,203,204,205]. Fungi used to cause several diseases in domestic animals by producing mycotoxins. Gibson et al. developed methods of immobilization of mycotoxins, such as aflatoxin B1 (AfB1) and ochratoxin A (OTA) by modified nanodiamond substrates through various mechanistic approaches, including carboxylation, hydrogenation, and hydroxylation [206].

Graphite is crystalline form of carbon. Extensive studies have been conducted human cell lines and the rodent system. However, the use of graphite in veterinary animals is limited. Very long back, Parvongnukul and Lumb (1978) used porous polytetrafluoroethylene-graphite coating for the long-term stabilization of total hip prostheses in goats [207]. The results from this study showed that effective stabilization in goats. Graphite-furnace atomic absorption spectroscopy used to determine the concentration of heavy metals and also blood selenium concentrations of goats and cattle [208,209], lead and cadmium concentrations in goat, cow, sheep, and buffalo milks [210].

Graphene is composed of sp^2^-hybridized carbon atoms, compared with carbon nanotubes, graphene-based materials can provide a larger surface area and better dispersibility in most solvents and it has attractive electronic, thermal, optical, and mechanical properties [211,212]. Graphene is a promising candidate for various biomedical applications due its versatility nature. Nanographene used as cellular imaging and drug delivery [213]. Reduced graphene oxide coated polydimethylsiloxane film as an optoacoustic transmitter for high pressure and high frequency ultrasound generation, which is used both human and veterinary medicine to treat and diagnose the diseases [214]. For instance, Ye et al. investigated the effect of GO of potential antiviral activity against pseudorabies virus (PRV, a DNA virus) and porcine epidemic diarrhea virus (PEDV, an RNA virus) [215]. Results from this showed that GO significantly suppressed the infection of PRV and PEDV for a 2-log reduction in virus titers at non-cytotoxic concentrations. The experiment was conducted to investigate the antimicrobial proprieties of three different graphene materials (pristine graphene (pG), graphene oxide (GO), and reduced graphene oxide (rGO) against the food-borne bacterial pathogens *Listeria monocytogenes* and *Salmonella enterica*. Among these three different nanomaterials, GO exhibited stronger effect at lower concentration than other nanomaterials [216].

**Figure 8:**
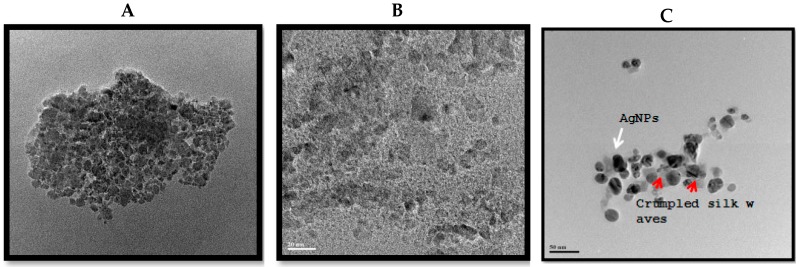
TEM images of (**A**) GO, (**B**) rGO, and (**C**) rGO–Ag nanocomposite. TEM images of fields were used to measure GO, rGO, and rGO–Ag particle.

**Figure 9:**
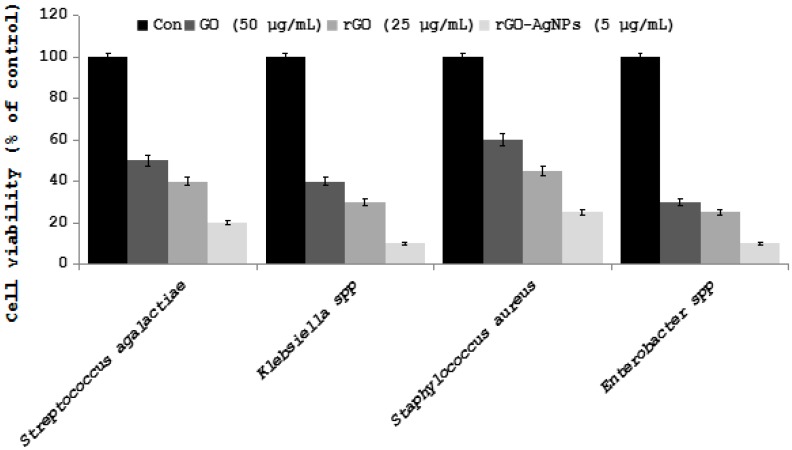
Effect of graphene oxide (GO), reduced graphene oxide (rGO), and GO-Ag on cell survival of *Staphylococcus agalactiae*, *Klebsiella* spp., *Staphylococcus aureu* and *Enterobacter* spp.

First fullerenes were discovered in 1985 and its attractive properties of physical, chemical, and biological yielded intense applications in both human and veterinary medicine. Fullerene molecules are composed entirely of carbon, in form of a hollow sphere, ellipsoid, or tube. Spherical fullerenes are also referred to as buckyballs. An important property of C60 molecule is its high symmetry [217]. Fullerenes (C60) and their derivatives have potential antiviral activity against HIV [218,219], semliki forest virus (SFV, Togaviridae), or vesicular stomatitis virus (VSV, Rhabdoviridae) [220]. Unformulated oligodeoxynucleotides (ODN) containing CpG motifs (CpG-ODN) are able to stimulate the innate immune system against a variety of bacterial, viral, and protozoan infections in a variety of vertebrate species. Ovo delivery of unformulated CpG-ODN was able to significantly protect neonatal broiler chickens against Escherichia coli or Salmonella Typhimurium infections [221]. Carbon nanotubes (CNTs) are made up of allotropes of carbon with a cylindrical nanostructure. Due to unique chemical and electromechanical properties of multiwalled carbon nanotubes (MWCNTs) are ideal candidates for the development of drug delivery platforms and effective antibacterial agents. The nanotubes can easily penetrate into bacterial cell membranes and they act as potential and selective antibacterial agents [222]. Hybrid nanomaterials composed of molybdenum disulfide nanosheets (MoS2) coated on functionalized multiwalled carbon nanotubes (f-MWCNTs) used the determination of chloramphenicol (CAP) in in food samples, like milk, honey, and powdered milk [223]. Antiviral drugs are difficult to enter into gastrointestinal tract, skin, and cell, therefore functionalized single-walled carbon nanotubes (SWCNTs) were selected as a drug carrier to carry antiviral drug for the treatment of viral diseases. The results show that SWCNTs has potential antiviral activity against reovirus [224]. To determine the effect of various carbon nanoparticles on bacteria causing mastitis in cows, we prepared graphene oxide, reduced graphene oxide, and silver nanoparticles graphene oxide nanocomposites (Figure 8), and the effect was examined in various pathogenic bacteria causing mastitis, and is displayed in figure (Figure 9) [225,226].

*Streptococcus agalactiae*, *Klebsiella* spp., *Staphylococcus aureu*, and *Enterobacter* spp. cells were incubated with GO (100 µg/mL) rGO (50 µg/mL), and rGO-Ag (10 µg/mL). Bacterial survival was determined at 24 h by a CFU count assay. The experiment was performed with various controls including a positive control (GO, rGO, rGO-Ag, and NB without inoculum) and a negative control (NB and inoculum without GO, rGO, and rGO-Ag). The results are expressed as the means SD of three separate experiments, each of which contained three replicates. Treated groups showed statistically significant differences from the control group according to the Student’s *t*-test (*p* < 0.05).

## 3. Role of Nanomaterials in Animal Health and Production

Nanotechnology has opened up new era in various area of research interest biotechnology, biomedical sciences; veterinary and animal sciences by providing new, small scale tools and materials that are beneficial for living organisms. The varieties of nanomaterials that are used for disease diagnosis, treatment, drug delivery, animal nutrition, animal breeding, reproduction and value addition to animal products, and finally food safety by using liposomes, polymeric nanoparticles, dendrimers, metallic nanoparticles, carbon nanoparticles, quantum dots, carbon nanotubes, magnetic nanoparticles, and fullerenes [227]. Recently, the nanotechnology contributes to the development of non-toxic antimicrobial agents to overcome antibiotic resistance through the use of devastating dose of antibiotics used for against various pathogens causing chronic infections to livestock, including *Brucella*, *Mycobacterium bovis*, *Streptococcus aureus*, and *Rhodococcus equi* [227]. Furthermore, the use of nanomaterials as antiviral agents, rapid and sensitive detection of viruses in veterinary medicine seems to be an important player for animal health and reproduction. Nanobiosensors are very sensitive for environmental monitoring and clinical diagnostics and also it has been used for reproductive management, such as detection of oestrus, hormone levels, and metabolites profiles [228,229]. Nanomaterials could be used for the cryopreservation of gonadal tissues, sperm, oocytes, and embryos, which are very essential in animal reproduction [230]. Finally, nanomaterials are being used in food technology including meat and meat products free from contaminations [231]. The nanomaterials would facilitate develop products and processes for animal health and production in line with economic, social, and environmental valuable materials to challenge the animal reproduction.

Recently, quantum dots have been explored to improve understanding of mammalian spermatozoon and oocyte movement and their interactions in a different physiological setting [91]. Biocompatible and self-illuminating inorganic nanoparticles show much interest in the field of theriogenology. For instance, quantum dots have been used as the real-time tracking ability of bioluminescent resonance energy transfer-conjugated quantum dot (BRET-QD) nanoparticles in vitro, in situ, and ex vivo using pig male gametes (*Susscrofa domesticus*) [232,233]. Further engineered nanoparticles with fluorescent probes used to visualize the molecular and cellular events during fertilization, in a similar way to fluorescent proteins, but at greater tissue depths [233,234].

## 4. Application of Nanosensors in Veterinary Medicine

Recently, the development and application of nanosensors getting immense interest due to several biomedical applications, such as measurement, include a huge number of simple and complex molecules, physical quantities such as pressure, force, displacement and flow, and electrical and magnetic phenomena arising from the heart, brain, muscles, and nerves [234]. Nanosensors are miniature devices that can diagnose biological material or tissue samples. Nanosensors have been used as invasive measurement to monitor important physiological variables, such as blood gases, ions, metabolites, blood pressure, and blood flow [235]. At present, sensors being developed based on variety of nanoparticles, NPs, such as nanoshells, nanowires, carbon nanotubes (CNT), and quantum dots (QDs), silver nanoparticles (AgNPs), and gold nanoparticles (AuNPs). Nanoparticle sensors are being used to measure O_2_, a number of ions, free radicals, and electric and magnetic fields [236]. Nanoparticles based sensors have been used in several biomedical applications in veterinary medicine, including animal health and other areas of animal production, particularly the delivery of controlled amounts of drugs into the beverage of breeding animals, prevention of bovine tuberculosis, the controlled release of injectable poorly water-soluble drugs, and as destroyer of pathogens [237].

Nanosensors and MEMS technologies provide excellent opportunities as gas sensors for the agri-food sector and also to monitor temperature, pressure, and other processing parameters [238]. Applications of nanosensors opened a new avenue ranging from whole body monitoring to diagnosing various diseases due to their unprecedented sensitivity. Majorly, nanosensors are working based on two detection principles, such as catalytic and affinity sensing. Catalytic sensors utilize enzymes, cells, tissues/organelles and microorganisms as the recognition agent. Affinity sensors are those that utilize whole antibodies, antibody fragments, nucleic acid/aptamers, receptors, lectins, phages, novel engineered scaffold derived bonding proteins, molecular imprinted polymers, plastic antibodies, and synthetic protein binding agents as the recognition agent [239]. Nanosensors have major role in veterinary sciences, they use very small amount of a chemical contaminant, virus, or bacteria, which is helpful for agriculture and food systems that in return improves the feedstock [240,241].

Nanosensors are miniature devices, which can identify samples that use biological material or tissue based on biorecognition element, which is immobilized on the surface of physicochemical transducer. Various types of nanosensors to utilze in the area of animal sciences food inspection, detection of cations, anions, and organic compounds in food, various aptamers for detection of pesticides, antibiotics, heavy metals, microbial cells and toxins, and also in feed and nutrient components, intelligent packaging, and quick detection systems [242]. Sensors are being used for various applications in animal health care to diagnose some disease spreading of viruses and microbial pathogens and also it can prevent death of animals. For instance, sensors and wearable technologies can be implanted on animals to measure body temperature, antibiotic detection, detect their sweat constituents, observation of behavior and movement, and stress in animals [243]. Sensors frequently used to monitor clinically important β-hydroxybutyrate BHBA to provide early diagnosis of Subclinical Ketosis (SCK) is essential for management of dairy cattle health. Peled et al. [244] detected *Mycobacterium bovis* in infected cattle via breath, which allows for real-time cattle monitoring. However, the use of sensors in veterinary medicine is limited because the biological sensing element is affected by different factors, including environmental factors and type of molecules.

## 5. Nanotechnology

Nanotechnology is an important emerging industry with a projected annual market of around one trillion US dollars by 2015. It creates novel materials with a variety of useful functions, including many that could be exceptionally beneficial in medicine. However, concerns are growing that it may have toxic effects, particularly damage to the lungs. The application of nanotechnology in medicine need special attentions is required related to the toxicology of nanoparticles and nanostructures. Therefore, exclusive nanotoxicology studies are warranted, particularly the subcategory of toxicology [245,246]. Classical categories are required for toxicological risk assessment of the use of nanoparticles, including hazard identification, hazard characterization, exposure assessment, and risk calculation Luther (2004). Since there are practically no toxicology studies available on the emerging applications of nanotechnology in medical technology, therefore studies are urgently required to address toxicological risks of the application of nanotechnology in medical technology. Furthermore, there is a lack of knowledge on the fate of ingested nanoparticles in human body and it is essential to investigate routes of exposure and also it is important to know about basic knowledge of their absorption, distribution, metabolism, and excretion. Finally, the implementation of a risk management strategy is required for all medical products using nanoparticles for all medical technology applications.

## 6. Conclusions and Future Perspective

Recent advances in nanotechnology is providing better opportunity for the development of novel nano-drug and delivery systems using non-toxic nanoparticles, such as liposomes, polymeric nanoparticles, dendrimers, and metal nanoparticles. Each type of nanoparticles has peculiar properties and these are used to enhance the therapeutic indices of the incorporated drugs in variety of ways, including bioavailability, retention time, protect the entrapped agent from the internal body environment, and sustained release. Among several different types of nanoparticles, liposomes are highly modifiable and they can be studied easily through their surface characteristics. The micellar nanoparticles are known to include greater loading capacity and superior stability, and also the use of MNPs is considered to be safer for parenteral administration. Dendrimers are monodisperse macromolecules combining with unique characters, these highly functionalized materials enable drug incorporation into the core of the molecule and drug complexation and conjugation on the surface, and also it has the capacity to carry a large amount of genetic material for efficient transport of DNA into the nucleus, it has immense benefits in animal production. Polymeric nanoparticles are highly stabile when in contact with biological fluids and their nature allows for controlled drug release, therefore polymeric nanoparticles have been used in several applications, including biological markers, imaging, healthcare products, pharmaceuticals, drug-delivery systems, as well as in detection, diagnosis, and treatment of various types of diseases in domestic and veterinary animals.

Recent years, metallic nanoparticles have become a center of attraction in nanomedicine due to their sizes, shapes, and ability to act as imaging and therapeutic drug scaffolds, as well as due to their intrinsic physicochemical unique properties. Further metallic nanoparticles explored intensively to variety of biomedical applications as “nanotheranostics”. Metallic nanoparticles act as synthetic platforms for a multimodal imaging approach to diagnosis and treatment of degenerative diseases, including cancer in human being and also extensively implemented in Veterinary Medicine as diagnostics, nanocarrier, therapeutic, and imaging agents. Targeted delivery of drugs by different metallic nanoparticles for veterinary infections provides great promise for both short-term and long-term treatment strategy. Critically, to realize the bright future of Veterinary Medicine as a whole, further detailed pre-clinical investigations are urgently needed in order to use all of the tools derived from variety of nanomaterials towards becoming a useful tool for various diagnosis and treatment in veterinary animals, like human being. Furthermore, it requires continuous and rigorous investigation into the short-term and long-term effects in vivo of such materials and a complete mechanistic understanding on a molecular scale to predict further hazards to living systems, as well as the environment. In Veterinary Medicine, it is very important to use very extensively nanoparticle mediated drug delivery to decipher a great potential to direct the drug more efficiently to the particular target site and also to overcome some of the biological barriers. The application of nanotechnology in animal production is still in its early stages, however many areas, including nutrition, biocidal, remedial, and reproductive studies are warranted.

Regenerative medicine is foremost important application of nanotechnology that can transform the designing of grafts and scaffolds for tissue regenerative properties in tissue engineering. When we looked at the status of regenerative medicine in Veterinary Medicine is countable. In response to this, international regulation of Veterinary Regenerative Medicine is underway. However, the development of ideal nanomaterials that are capable of sending signals to the diseased or damaged cells and tissues to trigger the regeneration process still remains a challenge. Similarly, the safety of animals in terms of the use of nanomaterials in regenerative medicine is a matter of considerable concern, because this field is still in its nascent stage. Uses of veterinary animals are increasingly recognized as critical translational models of human diseases. Prior to go to complete applications in Veterinary Medicine, studies on the beneficial/toxic effect of these nanomaterials should be carried out in great detail. Finally, to understand the underlying mechanisms of cell-biomaterial interactions at the nanoscale level, and to be able to translate the findings from bench to bedside, close collaboration between the scientists and veterinary clinicians is of utmost importance. Finally, advances in Veterinary Regenerative Medicine could offer relevant sources for studying human disease and also it could offers a venue for dissemination of information about veterinary therapeutics that can accelerate translational medicine. Furthermore, the studies are essential in the animal production industry and particularly to identify the gaps between knowledge and applications.
